# Endothelial Cell-Derived Exosomes Inhibit Osteoblast Apoptosis and Steroid-Induced Necrosis of Femoral Head Progression by Activating the PI3K/Akt/Bcl-2 Pathway

**DOI:** 10.1155/2024/3870988

**Published:** 2024-05-10

**Authors:** Jie Sun, Chen Yao, Wanxin Luo, Xingyu Ge, Wenjie Zheng, Chi Sun, Yafeng Zhang

**Affiliations:** ^1^Department of Orthopaedics, Affiliated Hospital of Nantong University, Nantong, Jiangsu, China; ^2^Research Centre of Clinical Medicine, Affiliated Hospital of Nantong University, Nantong, Jiangsu, China; ^3^Department of Geriatrics, Affiliated Hospital of Nantong University, Nantong, Jiangsu, China

## Abstract

The aim of the study was to investigate the therapeutic potential of exosomes secreted by endothelial cells (EC-exos) on steroid-induced osteonecrosis of femoral head (SNFH). First, we successfully obtained EC-exos through differential centrifugation. Then, the effects of EC-exos on mouse embryo osteoblast precursor (MC3T3-E1) cells under high concentration of dexamethasone (Dex) were analysed *in vitro*, which included cell migration, viability, and apoptosis. *In vivo*, a SNFH rat model was successfully established and treated with EC-exos. Micro-computed tomography (micro-CT) and haematoxylin and eosin (H&E) were used to observe femoral trabeculae. Our *in vitro* results showed that EC-exos improved cell viability and migration of osteoblasts and reduced the apoptotic effect of high concentration of Dex on osteoblasts *in vitro*. Phosphoinositide 3-kinase (PI3K)/Akt/Bcl-2 signalling pathway was activated in MC3T3-E1 cells under the response to EC-exos. *In vivo*, increased bone volume per tissue volume (BV/TV) (*p*=0.031), trabecular thickness (Tb.Th) (*p*=0.020), and decreased separation (Tb.Sp) (*p*=0.040) were observed in SNFH rats treated with EC-exos. H&E staining revealed fewer empty lacunae and pyknotic osteocytes in trabeculae. The expression of Bcl-2 and Akt in EC-exos group was significantly increased in trabeculae tissue. Overall, our finding indicated that EC-exos could attenuate SNFH by inhibiting osteoblast apoptosis via the PI3K/Akt/Bcl-2 pathway.

## 1. Introduction

Osteonecrosis of the femoral head (ONFH) is a common disease, with an incidence in US of 10,000 to 20,000 new cases annually [1]. ONFH affects millions of people around the world [2], caused by numerous traumatic or nontraumatic factors. The nontraumatic factors include excessive weight, alcohol abuse, and long-term use of glucocorticoids (GC). Among these, steroid-induced osteonecrosis affects a large number of patients, especially in the younger population [5].

Until now, the pathogenesis of steroid-induced necrosis of femoral head (SNFH) has not been clear. According to previous studies, SNFH is an irreversible disease associated with blood vessel damage and imbalanced bone homeostasis [6, 7]. SNFH often causes hip pain, which seriously affects the quality of life of patients, and even leads to disability in the later stage. A total hip arthroplasty (THA) is required to resolve patients' pain and restore mobility in the late pathological stage of the disease [8]. These factors indicated that early prevention of SNFH is very important.

With a diameter range of 40–150 nm, exosomes (exos) are microvesicles that contribute to intercellular communication [9]. Exos include proteins, microRNAs, and mRNAs, varying according to the cell type [10]. During the past few years, exos have attracted much attention as potential treatments for many diseases [11–13]. For example, exos secreted by bone marrow mesenchymal stem cells (MSC-exos) have been reported to treat severe acute respiratory distress both in mouse model and in clinic research [14, 15]; exos secreted by adipose cells and umbilical cord cells have been reported to promote wound healing in diabetes rats [16, 17]. In addition, studies over the past few years have also shown that endothelial cell-secreted exos (EC-exos) contain multiple bioactive components like miR-155 and miR-125-3p, which may regulate cell growth and migration, as well as vascular development [18]. A recent study revealed that EC-exos have natural bone-targeting properties and could mitigate *in vitro* osteoclast activities and prevent osteoporosis in ovariectomized mice [19]. Certain studies have also confirmed detrimental changes of vascular ECs during SNFH occurred [20, 21]. In this case, we developed the idea of using exos derived from normal (undamaged) vascular endothelial cells to prevent or treat SNFH. In addition, the effects of exos secreted by endothelial cells on SNFH have not been reported.

This study aimed to investigate the effects of EC-exos on the migration, viability, and apoptosis of MC3T3-E1 cells treated with high concentration of dexamethasone (Dex) *in vitro* and then evaluate the therapeutic effect of EC-exos on a rat model of SNFH *in vivo*.

## 2. Materials and Methods

### 2.1. Cell Culture

Mouse brain microvascular ECs (bEnd.3) and mouse embryo osteoblast precursor (MC3T3-E1) cells (iCell Bioscience Inc., Shanghai, China) were cultured in high-glucose Dulbecco's Modified Eagle Medium (DMEM) with 1.5 g/L NaHCO_3_ (iCell Bioscience Inc., Shanghai, China) and Minimum Essential Medium (MEM; HyClone, Logan, USA), respectively, with foetal bovine serum (FBS, 10%; Gibco, Carlsbad, USA) and penicillin/streptomycin (pen/strep, 100 U/mL and 100 *μ*g/mL; Gibco, USA) at 37°C in 5% CO_2_. bEnd.3 cells were used to prepare the exosomes, and the MC3T3-E1 cells were used for the *in vitro* model. MC3T3-E1 cells were divided into four groups: group 1, control group; group 2, EC-exos group, adding 50 *μ*g/ml EC-exos; group 3, Dex group, adding 10^−5^ M dexamethasone (D4902, Sigma-Aldrich, USA); group 4, Dex + EC-exos group, adding 10^−5^ M dexamethasone and 50 *μ*g/ml EC-exos.

### 2.2. Identification of Exos *In Vitro*

The bEnd.3 cells were switched to serum-free DMEM at 80% confluency. After 24 h, the supernatant was collected to obtain the exos through differential centrifugation (300 × *g* for 10 min, 1000 × *g* for 10 min, and 16500 × *g* for 30 min) at 4°C, passed through a 0.2 *μ*m filter (Millicell, Germany), and then ultracentrifuged twice (100,000 × *g* for 90 min) at 4°C. After an evaluation by transmission electron microscopy (TEM) and nanoparticle tracking analysis (NTA), the total protein of the exos was identified using a BCA kit (Beyotime, Shanghai, China), and western blotting was performed with antibodies against CD9 (SAB4503606; Sigma, USA), CD63 (sc-5275; Santa Cruz, USA), TSG101 (SAB5700757; Sigma, USA), and CD31 (11265-1-AP; Proteintech, China), endothelial cells' protein as a control after adjusting the concentration with a BCA kit.

### 2.3. Uptake of Exosomes

The EC-exos were analysed by the cellular uptake assay using the PKH-26 labelling kit (MINI26-1KT, Sigma, USA). At room temperature, the resuspended exos pellets (0.5 *μ*g/*μ*L) were incubated with PKH-26 dye (4 *μ*L) diluted in Diluent C (300 *μ*L) for 5 min, which was then mixed with FBS (3 mL) and centrifuged at 100,000 × *g* to pellet the exos labelled with PKH-26. Next, MC3T3-E1 cells were seeded in 6-well plates (5 × 10^4^ cells/well), and 24 h later, cells were incubated with the labelled exos for 2 h, then fixed in 4% paraformaldehyde (PFA) for 30 min at room temperature. The cell nuclei were stained with DAPI (Sangon Biotech, Shanghai), and the MC3T3-E1 cells were evaluated by fluorescence microscopy ((BX41, Olympus, Tokyo, Japan) (PKH-26: *λ*_ex_ 551 nm; DAPI: *λ*_em_ 567 nm).

### 2.4. Scratch Assay

MC3T3-E1 were seeded in 6-well plates (5 × 10^4^ cells/well) and culture medium was replaced with serum-free medium when the cells reached 90–100% confluency. A scratch wound was created in the cell monolayer using a 100-*μ*L pipette tip. After washing the cells with PBS to remove cell debris, the cells were treated according to the different methods of cell grouping mentioned above: group 1, control group; group 2, EC-exos group, adding 50 *μ*g/ml EC-exos; group 3, Dex group, adding 10^−5^ M dexamethasone; group 4, Dex + EC-exos group, adding 10^−5^ M dexamethasone and 50 *μ*g/ml EC-exos. Light microscopy images were taken at 0 and 24 h after wounding.

### 2.5. Transwell Assay

Selected in the logarithmic growth phase, MC3T3-E1 cells were suspended at 1 × 10^4^ cells/mL with culture medium containing 10 g/L bovine serum albumin (BSA, Thermo Fisher, USA). Each upper chamber contained 200 *μ*L of cell suspension, and each lower chamber contained 500 *μ*L of medium containing 10% FBS. The culture medium in lower chamber was treated according to the different methods of cell grouping mentioned above: group 1, control group; group 2, EC-exos group, adding 50 *μ*g/ml EC-exos; group 3, Dex group, adding 10^−5^ M dexamethasone; group 4, Dex + EC-exos group, adding 10^−5^ M dexamethasone and 50 *μ*g/ml EC-exos. The transwell plate was incubated at 37°C in 5% CO_2_ for 24 h. The cells were then fixed with 95% ethanol, stained by crystal violet (C0775, Sigma, USA), and imaged for cell counts under an inverted microscope (FKX41SF; Olympus, Tokyo, Japan).

### 2.6. Cell Counting Kit 8 (CCK-8) Assay

Cell proliferation was estimated using the CCK-8 assay (CK04, Kumamoto, Japan). Cells were seeded into 96-well plates (5,000 cells/well with 100 *μ*L complete medium), and the blank did not contain cells. The cells were treated according to the different methods of cell grouping mentioned above: group 1, control group; group 2, EC-exos group, adding 50 *μ*g/ml EC-exos; group 3, Dex group, adding 10^−5^ M dexamethasone; group 4, Dex + EC-exos group, adding 10^−5^ M dexamethasone and 50 *μ*g/ml EC-exos. At 12, 24, 36, and 48 h, the cells in each well were treated with 10 *μ*L CCK-8 solution and incubated at 37°C for 1 h. Measuring the absorbance at 450 nm, the survival/proliferation of the cells was calculated by subtracting the optical density (OD) of the blank from the OD of the cells.

### 2.7. Flow Cytometry Assay

The apoptotic effect of EC-exos on MC3T3-E1 cells in the high Dex level was estimated using annexin V-FITC/PI double staining kit (6592S, Cell Signaling Technology, Danvers, MA, USA). Briefly, the cells were treated according to the different methods of cell grouping mentioned above: group 1, control group; group 2, EC-exos group, adding 50 *μ*g/ml EC-exos; group 3, Dex group, adding 10^−5^ M dexamethasone; and group 4, Dex + EC-exos group, adding 10^−5^ M dexamethasone and 50 *μ*g/ml EC-exos, and harvested after being cultured in serum-free medium for 96h and resuspended in 96 *μ*L of annexin V binding buffer. The cells were subsequently incubated with 1 *μ*L of annexin V-FITC and 12.5 *μ*L of propidium iodide (PI) for 10 minutes on ice in the dark. Samples were then diluted to 250 *μ*L with annexin V binding buffer and analysed by flow cytometry.

### 2.8. Western Blotting

Equal amounts (100 *μ*g) of protein were separated by sodium dodecyl sulphate-polyacrylamide gel electrophoresis (SDS-PAGE) and transferred to polyvinylidene difluoride (PVDF) membranes. The membranes were blocked with 5% BSA and then incubated with primary antibodies at 4°C overnight. Next, membranes were incubated with horseradish peroxidase-conjugated secondary antibodies (1 : 5000, 7076S, Cell Signaling Technology, USA) for 2 h at room temperature. The primary antibodies used were anti-Bax (1 : 1000, 50599-2-Ig, Proteintech), anti-Bcl-2 (1 : 1000, 26593-1-AP, Proteintech), anti-Akt (1 : 1000, 60203-2-Ig, Proteintech), anti-p-Akt (1 : 1000, 66444-1-Ig, Proteintech), anti-PI3K (1 : 1000, 20584-1-AP, Proteintech), and anti-p-PI3K (1 : 1000, 17366S, Cell Signaling Technology, USA). The blots were visualized using an enhanced chemiluminescence kit (Millipore Corporation, USA).

### 2.9. Animal Model

Overall animal experimental designs and schemes were approved by the Institutional Animal Care and Use Committee (IACUC) in the School of Medicine, Nantong University (No. S20200723-902). A rat model of SNFH was established for this study using female Sprague–Dawley (SD) rats (*n* = 32) weighting 200–220 g and methylprednisolone (MPS, 20 mg/kg, HY-B0260, MedChemExpress, USA). SD rats were purchased from the Laboratory Animal Centre of Nantong University. The rats were bred in the institutional animal house and kept at 22°C under a 12 h light/12 h dark cycle, received standard rat chow and water *ad libitum*. After one week of adaptive breeding, rats were randomly divided into four groups (*n* = 8/group): (1) control group—intraperitoneal injection with 2 mL saline every Monday, Wednesday, and Friday, followed by tail vein injection with 200 *μ*L PBS; (2) EC-exos group—intraperitoneal injection with 2 mL saline every Monday, Wednesday, and Friday, followed by tail vein injection with 100 *μ*g EC-exos dissolved in 200 *μ*L PBS; (3) Dex group—intraperitoneal injection with MPS dissolved in 2 mL saline every Monday, Wednesday, and Friday, followed by tail vein injection with 200 *μ*L PBS; and (4) Dex + EC-exos group—intraperitoneal injection with MPS dissolved in 2 mL saline every Monday, Wednesday, and Friday, followed by tail vein injection with 100 *μ*g EC-exos dissolved in 200 *μ*L PBS. The entire procedure was repeated for 4 weeks.

All rats were sacrificed in the sixth week. The left femoral heads of rats in the control, Dex, and Dex + EC-exos groups underwent micro-computed tomography (CT) analysis, while the right femoral heads were processed for haematoxylin and eosin (H&E) staining, TUNEL assay, and immunohistochemistry (IHC) analysis. Lung, liver, spleen, kidney, and heart tissues of the control and EC-exos groups were collected to assess the biosafety of EC-exos by H&E staining.

### 2.10. Micro-CT Scanning

Rats in the control, Dex, and Dex + EC-exos groups were sacrificed, and the femoral heads were collected and fixed in formalin for micro-CT analysis (SkyScan-1176 micro-computed tomography system, Bruker Micro-CT, Kontich, Belgium) at 9 *μ*m per pixel. The generated coronal, sagittal, and transverse images by DataViewer (Bruker Micro-CT) were used to analyse trabecular thickness (Tb.Th), separation (Tb.Sp), number (Tb.N), and bone volume per tissue volume (BV/TV).

### 2.11. H&E and IHC Staining

The decalcified femoral heads and other tissues were embedded in paraffin and used for histological observations following H&E staining. First, the decalcified femoral head tissues were decalcified for 4 weeks. 5 *μ*m sections were cut on a paraffin slicing machine and rehydrated. After blocked, tissue sections were incubated with primary antibodies against p-Akt (1 : 200, 66444-1-Ig, Proteintech), Bcl-2 (1 : 200, 26593-1-AP, Proteintech), and Bax (1 : 200, 50599-2-Ig, Proteintech) overnight at 4°C, and then incubated with biotin-labelled secondary antibodies (1 : 1000, SA00004−2, Proteintech) at 37°C for 1 h. Finally, 3,3-diaminobenzidine (DAB, Sigma, St. Louis, MO, USA) and haematoxylin counterstained and the slides were sealed and observed with a microscope (Leica).

### 2.12. TUNEL Assay

Using a TUNEL kit (C10625, Thermo Fisher, USA), the sectioned specimens were digested with Proteinase K. Then, the terminal deoxynucleotidyl transferase (TdT) and Green fluorescent protein-2′-deoxyuridine-5′-triphosphate (GFP-d-UTP) were added into the labelling buffer. After that, specimens were labelled for 2 h at 37°C in the dark. Apoptotic cells identified by green fluorescence were counted in each unit area.

### 2.13. Statistical Analysis

The results were presented as mean ± standard deviation (SD). For the data from three or more than three groups, if the variance was uneven, log conversion or nonparametric test would be performed on the data. If the variance satisfied homogeneity, one-way analysis of variance was performed. Once the test was considered statistically significant, the comparison between any two pair of groups was made with Fisher's LSD test. In all tests, *p* value less than 0.05 was considered statistically significant. Student's *t*-tests were used to compare the data from two groups. A *p* value of less than 0.05 was regarded as statistically significant. All the statistical analyses were performed using SPSS software (version 13.0, USA).

## 3. Results

### 3.1. The Characterization of EC-Exos and Validation of Uptake by Osteoblasts

**Figure 1:**
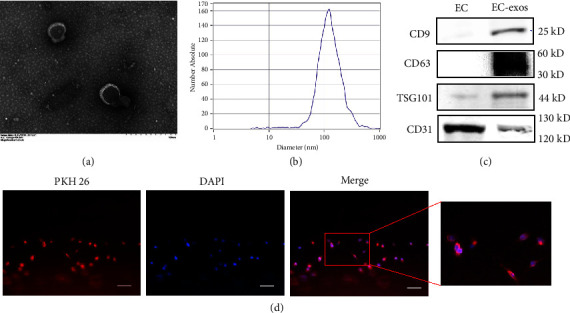
EC-exos characterization and the uptake by osteoblasts. (a) Representative image of exosomes secreted by endothelial cells by transmission electron microscopy. (b) Nanoparticle analysis of EC-exos. (c) Western blotting of exosomal markers CD9, CD63, CD31, and TSG101. (d) The uptake of exosomes in osteoblasts by PKH26 staining. Scale bar = 50 *μ*m.

To detect the characterization of extracted extracellular vesicles, TEM, NTA, and western blotting were performed. TEM was used to observe the specific morphology of exosomes. NTA was used to detect the average diameter of exosomes. Western blot was used to detect protein markers of exosomes. TEM showed that the EC-exos presented as cup-shaped vesicles with a diameter about 150 nm (Figure 1(a)). NTA showed that the particle size of exosomes was 132.3 ± 11 nm (Figure 1(b)). Western blotting revealed that the EC-exos surface biomarkers (CD9, CD63, CD31, and TSG101) were all positively expressed (Figure 1(c)). Images from the EC-exos uptake experiment confirmed that the EC-exos were taken up by the MC3T3-E1 cells (Figure 1(d)).

### 3.2. EC-Exos Facilitated Cell Viability and Migration of Osteoblasts under Dex *In Vitro*

**Figure 2:**
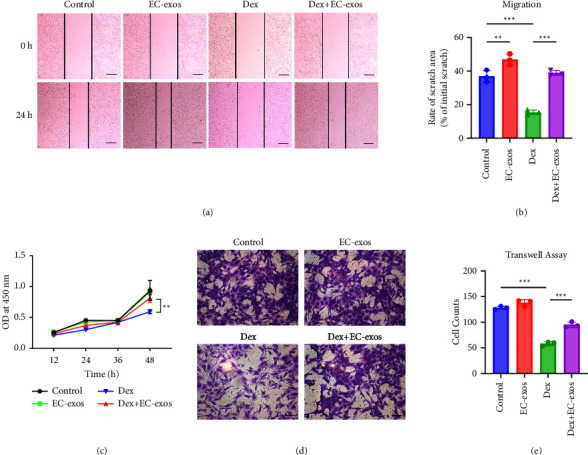
EC-exos facilitated the cell viability and migration of osteoblasts under Dex *in vitro*. (a) Scratch assay for evaluating the migration capacity of MC3T3-E1 cells at 0 and 24 h in different groups. Scale bar = 100 *μ*m. (b) Quantitative analysis of the migration area of MC3T3-E1 cells (*n* = 3). (c) The cell viability of different treatments on MC3T3-E1 cells were tested by CCK-8 assay. (d) The migration capacity of MC3T3-E1 cells was investigated by transwell assay in different groups. Scale bar = 100 *μ*m. (e) Quantitative analysis of cell migration by transwell assay (*n* = 3). ^*∗*^*p* < 0.05, ^*∗∗*^*p* < 0.01, ^*∗∗∗*^*p* < 0.001.

To detect cell viability of MC3T3-E1 cells in different groups, CCK-8 assay was used. As shown by the CCK-8 assay, EC-exos promoted the viability of MC3T3-E1 cells in Dex + EC-exos group compared with Dex group (*p* < 0.001) (Figure 2(c)). To test the migration ability of MC3T3-E1 cells in different groups, the scratching assay and transwell assay were used. The scratching assay showed that the cell migration of MC3T3-E1 cells was significantly decreased in Dex group compared with that in control group (*p* < 0.001). In contrast, the inhibitory effect of Dex was restored by EC-exos (*p*=0.010) (Figures 2(a), 2(b)). In addition, transwell assay also showed that Dex reduced the migration ability of MC3T3-E1 cells in Dex group compared with control group (*p* < 0.001) and EC-exos improved the migratory ability of cells treated with Dex (*p* < 0.001) (Figures 2(d), 2(e)).

### 3.3. EC-Exos Inhibited Osteoblast Apoptosis via the PI3K/Akt/Bcl-2 Signalling Pathway *In Vitro*

**Figure 3:**
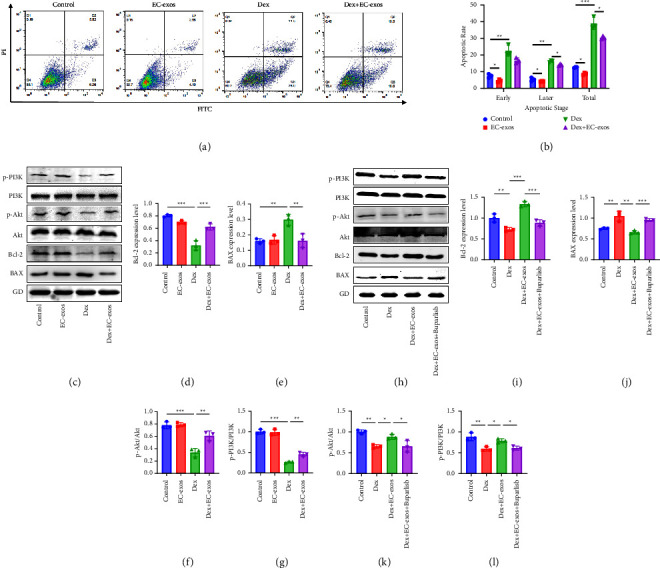
EC-exos inhibited osteoblasts apoptosis *in vitro*. (a) The apoptosis of MC3T3-E1 cells induced by Dex together with or without EC-exos was assessed through annexin V-FITC/PI double staining with flow cytometric analysis. (b) Quantitative analysis of the cell apoptosis of MC3T3-E1 cells (*n* = 3). (c) Western blotting of p-PI3K, PI3K, p-Akt, Akt, Bcl-2, and Bax in MC3T3-E1 cells exposed to Dex and co-treated with or without EC-exos. (d, e) The relative expression of Bcl-2 and Bax in C (*n* = 3). (f, g) The ratio of relative expression of p-PI3K/PI3K and p-Akt/Akt in C (*n* = 3). (h) Western blotting of p-PI3K, PI3K, p-Akt, Akt, Bcl-2, and Bax in MC3T3-E1 cells exposed to Dex + EC-exos and co-treated with or without PI3K inhibitor buparlisib. (i, j) The relative expression of Bcl-2 and Bax in H (*n* = 3). (k, l) The ratio of relative expression of p-PI3K/PI3K and p-Akt/Akt in H (*n* = 3). ^*∗*^*p* < 0.05, ^*∗∗*^*p* < 0.01, ^*∗∗∗*^*p* < 0.001.

Flow cytometry assay was to measure the apoptosis of MC3T3-E1 cells in different groups. The results of flow cytometry assay showed that Dex significantly increased the early, late, and final stages of apoptosis in Dex group compared with that in control group (*p* < 0.001), and the treatment of EC-exos was able to significantly reduce the apoptotic effect of Dex on MC3T3-E1 cells (*p* < 0.001) (Figures 3(a), 3(b)). Western blotting was used to measure the change of protein in PI3K/Akt/Bcl-2 signalling pathway (Figure 3(c)). The results showed that the expressions of the apoptosis-related protein, BAX, were increased in the Dex group and decreased in Dex + EC-exos group (*p* < 0.001), while the antiapoptosis-related protein, Bcl-2, was decreased in the Dex group and increased in Dex + EC-exos group (*p* < 0.001). EC-exos upregulates the expression of p-Akt and p-PI3K (Figures 3(c)–3(g)). After adding specific PI3K/Akt/Bcl-2 signalling pathway inhibitor, buparlisib (10 *μ*M, SJ-MX0283, China), the ability of EC-exos to inhibit steroid-induced apoptosis was impaired (Figures 3(h)–3(l)).

### 3.4. EC-Exos Showed Good Biocompatibility and Prevented the Progression of SNFH in Rat Model

**Figure 4:**
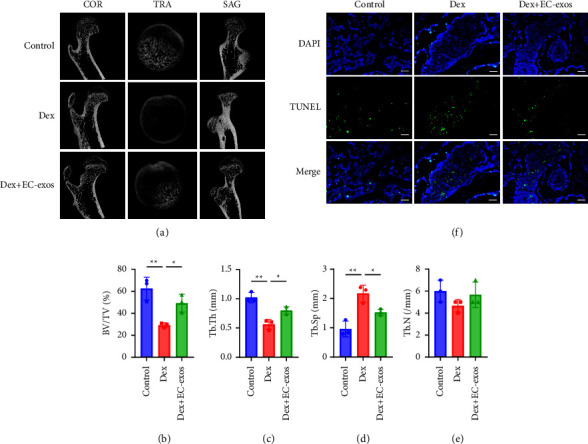
The improvement effects of EC-exos on the rat model of SNFH. (a) Three plane images including coronal section (COR), sagittal section (SAG), and transverse section (TRA) of the femoral head were reconstructed in the different groups. (b–e) Quantitative analyses of bone volume per tissue volume (BV/TV), trabecular thickness (Tb.Th), trabecular separation (Tb.Sp), and trabecular number (Tb.N) in the different treatment groups (*n* = 3). (f) The effects of EC-exos on the rat model were evaluated by TUNEL assay. Scale bar: 50 *μ*m. ^*∗*^*p* < 0.05, ^*∗∗*^*p* < 0.01, ^*∗∗∗*^*p* < 0.001.

To explore the therapeutic effects and biocompatibility of EC-exos, a rat model of SNFH was established. The micro-CT results showed higher rates of bone mineral loss and a greater cystic degeneration in the subchondral bone area of the femoral heads, as well as spare and thin trabecular bone in the Dex group compared to the control group. Meanwhile, part of the femoral neck showed compensatory bone sclerosis in the Dex group (Figure 4(a)). Importantly, these deteriorations were all mitigated in the Dex + EC-exos group. Increased BV/TV (*p*=0.031) and Tb.Th (*p*=0.020) were observed in the Dex + EC-exos group in comparison with the Dex group, while Tb.Sp was reduced (*p*=0.040). There is an upward trend of Tb.N in the Dex + EC-exos group in comparison with the Dex group (Figure 4(b)). The EC-exos demonstrated a rescue effect on bone deterioration and an osteogenic effect in this study. The antiapoptosis-promoting effects of EC-exos on the rat model of SNFH were evaluated by TUNEL assay. The results showed that the apoptotic characteristics in the trabecular bone in the Dex group were significantly reduced with the addition of EC-exos compared to the control group (Figure 4(f)).

**Figure 5:**
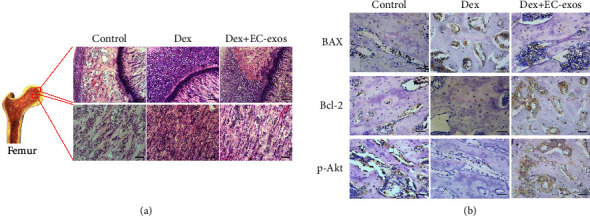
H&E staining and IHC staining of femoral heads in rat model with different treatments. (a) H&E staining of the femoral heads in rats receiving different treatments. Scale bar: 200 *μ*m. (b) IHC staining of p-Akt, Bcl-2, and Bax in samples from the different treatment groups. Scale bar: 50 *μ*m.

H&E staining revealed more empty lacunae and pyknotic osteocytes in the trabecular bone cavities, which led to a sparse trabecular bone structure in the Dex group. The marrow cavity showed typical osteonecrotic lesions in the femoral head, which were indicated by karyopyknosis and nuclear fragmentation surrounded by bone marrow cell necrosis (Figure 5(a)). The IHC analysis showed that the expression of p-Akt and Bcl-2 was increased in the tissues (Figure 5(b)).

**Figure 6:**
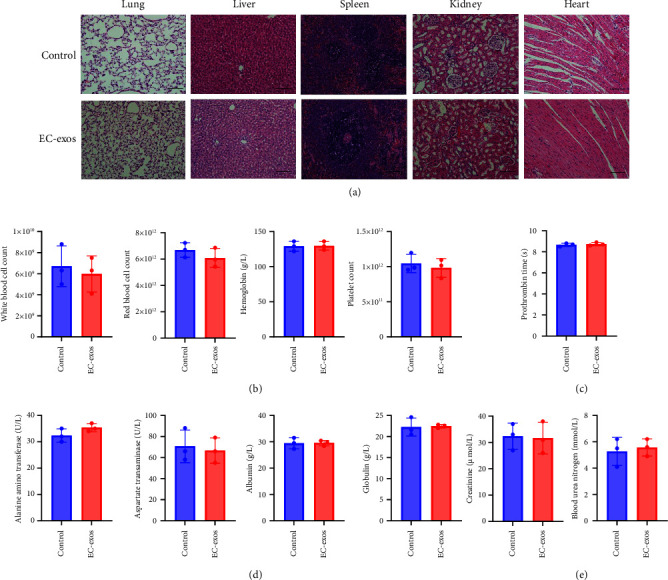
Verification of biological safety of EC-exos *in vivo*. (a) H&E staining of heart, liver, lung, kidney, and spleen in rat model without treat or treating with EC-exos only. Scale bar = 100 *μ*m. (b) Blood routine index at week 6 (*n* = 3). (c) Prothrombin time at week 6 (*n* = 3). (d) Liver function indicators including alanine aminotransferase (ALT), aspartate transaminase (AST), albumin (ALB), and globulin (GLB) at week 6 (*n* = 3). (e) Kidney function indicators including creatinine (Cr) and blood urea nitrogen (BUN) at week 6 (*n* = 3).

H&E staining of the lung, liver, spleen, kidney, and heart tissues of the EC-exos group showed no difference from the control group, indicating that the EC-exos had good biocompatibility (Figure 6(a)). In addition, we also measured blood routine index, prothrombin time, and liver function indicators including alanine aminotransferase, aspartate transaminase, albumin, globulin, kidney function indicators creatinine (Cr), and blood urea nitrogen in the rat, which injected 100 *μ*g EC-exos at week 6 (Figures 6(b)–6(e)). The results showed that there was no difference between the EC-exos group and the control group.

## 4. Discussion

Steroids are hormones commonly used in patients to improve immunity and reduce disease duration [22]. However, long-term use of steroids can cause damage or interruption to blood circulation, apoptosis of osteoblasts in the femoral head, and cell necrosis, which ultimately lead to SNFH characterized by hip pain and dysfunction [23, 24]. Patients in the later stages of SNFH experience the joint deformities and pain during movement, and they will eventually require the THA surgery [25]. Impaired cell viability, migration ability, and cell apoptosis are the key points of SNFH, which is induced by glucocorticoids (GC) [26]. High concentrations of GC have a strong inhibitory effect on angiogenesis, and it can reduce bone formation and increase bone resorption [7]. For osteoblasts, high concentrations of GC could increase oxidative stress levels and lead to apoptosis [27].

In recent years, numerous studies have described a beneficial interaction between blood vessels and bone homeostasis [28]. Blood vessel formation is an important part of bone formation, skeletal development, and the osteointegration process [29]. The surface of both endothelial cells and osteoblasts expresses vascular endothelial growth factor receptor 2 (VEGFR2), which integrates the processes of bone formation and angiogenesis [28, 30]. It had been reported that human umbilical vein endothelial cells (HUVECs) could stimulate osteoblast proliferation and vascular endothelial cells were usually combined with osteoblasts or MSC according to many researches of bone repair [31, 32]. Although the synergistic effects of osteogenesis and angiogenesis have been confirmed in many studies [26, 33, 34], while little research has focused on the therapeutic potential of exosomes released by endothelial cells for SNFH.

In this study, we measured the effects of EC-exos on MC3T3-E1 cells under high concentration of GC *in vitro*, which included cell migration, viability, and apoptosis. *In vivo*, a SNFH rat model was successfully established and treated with EC-exos. Micro-CT and H&E staining were used to observe femoral trabeculae. Our *in vitro* results indicated that the effects of EC-exos were to facilitate cell viability, increase migration ability, and reduce the apoptosis of MC3T3-E1 cells under high concentrations of GC. *In vivo* experiments demonstrated that the EC-exos attenuated the progression of SNFH. The micro-CT scans revealed that the EC-exos improved SNFH-related bone parameters, including BV/TV, Tb.Th, Tb.N, and Tb.Sp. The EC-exos mitigated the osteonecrosis and increased the amount of trabecular bone. The histological analysis showed that the EC-exos reduced the formation of necrotic tissue in the femoral head. The treatment effect we obtained is comparable to the treatment effect reported in previous literature [35, 36]. In addition, the biosafety assessment of the EC-exos was assessed following the injection of EC-exos in rats for six weeks. The H&E staining of the lung, liver, spleen, kidney, and heart tissues in the EC-exos group showed no difference from the control group. In addition, there was no difference between the EC-exos group and the control group in blood routine index, prothrombin time, and liver function indicators, including alanine aminotransferase, aspartate transaminase, albumin, globulin, kidney function indicators creatinine (Cr), and blood urea nitrogen. Those results indicated that the EC-exos had good biocompatibility.

According to the pathological characteristics of SNFH, the formation of sclerotic bone is one of the main characteristics in the middle stages of the disease [37]. Our micro-CT results in this study showed that the sclerotic bone area was reduced in the Dex + EC-exos group compared to the Dex group, indicating that the EC-exos could reduce the speed of sclerotic bone formation as well as prevent the pathological progress of SNFH. In addition, the TUNEL assay of the femoral head specimens showed that the increased apoptotic characteristics that were observed in the trabecular bone of the Dex group compared to the control were significantly reduced with EC-exos treatment.

The PI3K/Akt/Bcl-2 signalling pathway is important for the regulation of the cell proliferation, differentiation, and apoptosis [38, 39]. After receiving various signals at the cell membrane, PI3K can directly or indirectly activate Akt, and p-Akt can phosphorylate p53, nuclear factor-kappa B (NF-*κ*B), and caspase-3, which ultimately exert specific biological effects. Numerous previous studies have demonstrated a correlation between the PI3K/Akt signalling pathway and the antiapoptotic protein Bcl-2 [38, 40]. Consistent with the previous research, EC-exos increased the levels of p-PI3K/p-Akt and the expression of Bcl-2, thereby inhibiting cell apoptosis. Meanwhile, the IHC assay also showed that p-Akt and Bcl-2 were significantly present in the bone tissue, indicating that the PI3K/Akt/Bcl-2 signalling pathway plays an important role in the protective mechanism of EC-exos in SNFH.

## 5. Conclusion

In summary, the present study provided evidence that EC-exos can inhibit steroid-induced apoptosis of osteoblasts via the activation of the PI3K/Akt/Bcl-2 signalling pathway. The EC-exos can attenuate disease progression in a rat model of SNFH, which may be a potential therapeutic strategy for the clinical treatment of SNFH.

## Data Availability

The datasets used and/or analysed during the current study are available from the corresponding author upon reasonable request.

## Abbreviations

EC-exos: Exosomes secreted by endothelial cells
SNFH: Steroid-induced osteonecrosis of femoral head
Akt: Protein kinase B
Bcl-2: B-cell lymphoma 2
Bax: Bcl-2-associated X protein
PI3K: Phosphoinositide 3-kinase
Dex: Dexamethasone
BV/TV: Bone volume per tissue volume
Tb.N: Trabecular number
Tb.Th: Trabecular thickness
Tb.Sp: Trabecular separation
GC: Glucocorticoids
THA: Total hip arthroplasty
MSCs: Bone marrow mesenchymal stem cells
bEnd.3: Mouse brain microvascular ECs
MC3T3-E1: Mouse embryo osteoblast precursor
TEM: Transmission electron microscopy
NTA: Nanoparticle tracking analysis
MPS: Methylprednisolone
TUNEL: Terminal deoxynucleotidyl transferase-mediated dUTP-biotin nick end labelling.
